# The Study of Two Psychotherapy Approaches (Rogers Self Theory and Ellis Rational Theory) in Improvement of Bowen Self-differentiation and Intimacy

**Published:** 2014

**Authors:** Naser Yousefi, Mohammad Ali Kiani

**Affiliations:** 1 Assistant Professor, Department of Family Counseling, School Literature and Social Sciences, University of Kurdistan, Sanandaj, Iran.; 2 Department of Psychology, University of Kurdistan, Sanandaj, Iran.

**Keywords:** Intimacy, Person-Centered Therapy (PCT), Rational Emotive Behavior Therapy (REBT), Self-Differentiation

## Abstract

**Objective: **To compare the effectiveness of rational, behavioral and emotive therapy (REBT) and person-centered therapy (PCT) on self-differentiation and intimacy among divorce clients.

**Methods:** In quasi-experimental study, 42 divorce clients (both males and females) who presented to the Counsling Center of Sanandaj, Iran were sampled. They were categorized into three groups of PCT, REBT, and control group (each group contained 14 subjects). The recovery indices (dependent variables) employed were the subject of self-differentiation and intimacy, which were measured twice before and after intervention of Differentiation of Self Inventory-2 (DSI-2) and intimacy. The therapy involved 8 one-hour sessions. It was held twice a week and therapeutic effects were traced after 8 months.

**Results:** The results showed that REBT and PCT were effective on self-differentiation scale and intimacy. Also they were influential in recovery self-differentiation scale and intimacy follow up stage.

**Conclusion:** REBT and PCT were effective on self-differentiation and its subscales (Emotional reactivity, “I” position, Emotional cut off and Fusion with other) and general intimacy.

**Declaration of interest: **None.

## Introduction

Psychotherapy is aimed at strengthening the individual’s mental and emotional resources so that he can function more effectively. Two forms of psychotherapy are Person-Centered Therapy (PCT) and Rational-Emotive-Behavior Therapy (REBT) (1, 2). PCT is often called self-theory, non-directive counseling or Rogerian counseling. Rogers (3), who introduced this therapy, labeled it as“client-centered therapy”. Recently, Rogers labeled it as PCT. PCT was originated in the US. This approach stresses on the ability of clients to determine the issues important to them and to solve their problems (4). The most important quality of the counseling relationship is the establishment of warm, permissive and accepting climate that permits clients to explore their self-structure in relation to their unique experiences. The person-centered approach to counseling is based on a very positive view of human nature. The method utilizes active listening, reflection of feelings, clarification and facilitation (5).

Ellis (6) formulated REBT. He stated that he views humans as both rational and irrational (6). Emotional problems lie on illogical beliefs. Blame and anger are viewed as dysfunctional and irrational feelings (7). The rational-emotive-behavioral practitioner believes that no person is to be blamed for any thing he or she does, but each person is responsible for his or her behavior. Ellis has formulated a theory of personality identified as the A-B-C-D theory (8). When an individual has an emotional reaction point C (the emotional consequence), after some activating agent, event, or experience has occurred (point A), it is viewed as the result of the system (point B). A does not cause C, but the belief system that is held about A leads to C (8).

Self-differentiation, the most central concept in Bowen’s theory, has both intra-psychic and interpersonal dimensions. On an intra-psychic level, differentiation refers to the ability to distinguish emotional feelings from other intellectual processes. With the interpersonal level in mind, differentiation involves the capacity to develop a balance of autonomy while maintaining closeness with others. Intra-psychic dimensions of differentiation include emotional reactivity and difficulty in taking an “I” position, while interpersonal dimensions include emotional cut-off and fusion with others. These persons tend to engage in fusion with or emotional cut-off from others in most of their close relationships when under stress (9-13).

In interpersonal situations, poorly-differentiated persons are thought to engage in fusion or emotional cut-off in response to stress or overwhelming anxiety (14-16).

Researchers believe that low differentiation levels contribute to marital conflicts (17-19). In marital relationship, whenever the differentiation level falls low, fusion takes place between couples, leading to low marital quality and compatibility (13, 16, 17, 20-26).

Using a Philippine sample, Tuason and Friedlander (27) tested the cross-cultural applicability of Bowen’s theory and reported a significant influence of differentiation on anxiety, similar to the results from USA samples (28). Haber (29), for example, found that couples with higher levels of differentiation had lower levels of relationship conflicts. Another study of married couples also found a significant relationship between differentiation and marital satisfaction (30). In a similar vein, Skowron (31) found a positive correlation between differentiation and marital satisfaction, with husbands’ emotional cut-off scores particularly correlating with both husbands’ and wives’ marital satisfaction scoresnces.

Former studies have examined the relationship between differentiation of self level and subjective well-being (13, 32), mental health dimensions and symptoms of psychic disorders (33-35), marital adjustment (36), styles of marital relationship (21, 37-39), couple's compatibility (40), marital satisfaction (18, 41), cordiality and sexual satisfaction (42, 43).

Sternberg (44) believes that intimacy is a feeling creating closness, belonging and contact. Intimacy is the main and real need of human being and it is not just a desire.

The research showed that in high-commitment marriages, there is more intimacy and love and marital conflicts are less observed (45, 46).

Hefazati et al (47) and Sobhi (48) found that three components of excitement, intimacy and commitment are correlated with marital satisfaction. Mitchell et al. (49) in prediction of intimacy factors between couples in their study found that self-disclosure and emphatic responding are the most important behaviors in terms of intimacy of couples. It has been shown that couples do not have significant difference in five dimensions of intimacy, namely relationship orientation, caring, concern, sexuality and communication (50).

*What did this study explore?*

The present study attempts to answer this questions: Are REBT and PCT effective on self-differentiation and intimacy? 

## Materials and Methods

***Design***

The methodology used in this research involved a quasi-experimental design.

***Target Population***

All divorce clients who presented to the Counseling Center of Sanandaj, Iran, were considered as the target population.

***Sampling***

On-hundered and seventy-eight clients (woman and man) were selected through stratified random sampling method. Then 42 subjects (11 males and 31 females) were selected through random sampling method and were randomly assigned to three groups (14 subjects in each group): PCT group, REBT group, and control group.

***Measurement***

The sample was surveyed regarding self-differentiation and intimacy disorder diagnosis with Differentiation of Self Inventory-2 (DSI-2) and intimacy Scale 

***Interventions***

Two groups of clients (male and female) who complained of marital conflicts were exposed to two different therapeutic interventions:

Group one (PCT group): Subjects in this group were treated through Roger’s PCT procedure individually by a trained counselor in the Counseling Center. This method was offered in eight one-hour sessions. It was held two times a week. After eight months, therapeutic effects were traced. Manual of person-centered therapy:

Session1: According to Rogers, how we think and feel about ourselves (our sense of self-worth) is fundamental both to psychological health and to the likelihood that we can achieve goals and ambitions in life. Session 2: Positive regard: it is to do with how other people evaluate and judge us in social interaction. Session 3: Self-concept and congruence/incongruence: the self-concept consists of perceived self; how someone actually sees himself/herself, the respondent answers to: “Who am I? What do I think and feel? etc".. Session 4: Congruence:  the aim of PCT is to increase the client's congruence. Session 5: Unconditional positive regard: to create an atmosphere of psychological safety (warmth and acceptance) within the counselling relationship. Session 6: Acceptance: within the counselling relationship. Session 7: Empathic understanding: the person-cantered therapist should sense the client's world, their subjective experiences and perceptions, as if it were her or his own. Session 8: Self-actualization: it can be defined as a state of psychological fulfillment, including acceptance of self and others.

Group two (REBT group): In this experimental group, Elliis REBT method was implemented. This method was performed by an experienced and well-informed counselor in the Counseling Center. The therapy involved 8 one-hour sessions. It was held twice a week and therapeutic effects were traced after 8 months. Manual of REBT: Session1: Cognitive-affective-behavioral theory: irrational beliefs are beliefs that are unrealistic, illogical, and absolutist. It is a person’s irrational belief that leads to great anxiety, depression, shame, anger, guilt, not the event which he/she is experiencing. Session 2: Therapy: A-B-C-D-E. A: activating event: Ali asks Maryam if she would go out with him, and she replies that she is busy every Saturday night this year. Session 3: B. Irrational believes. Session 4: C. undesirable consequences: Eeotions: feelings of worthlessness, feel anxiousness, and depressed. Session 5: disputing irrational believes is it awful that she rejected me? How am I worthless because she refused me? Where is the evidence that no desirable woman will ever accept me? Session 6: E. emotional effects appropriate feelings: sorrow, frustration, disappointment, self-acceptance, and hopeness. Session 7: E. behavioral effects. Session 8: desired behaviors: improve myself, keep pursuing, ask someone else for dining out. 

***Ethics***

The clients were informed that this intervention is a part of the research. Besides the results would be useful for them, the items that are discussed in the therapy sessions are not discussed with any family member, even spouse without the satisfaction of the clients. In addition, their identity about the resutls of the questionnaire regarding intimacy would be considered confidential and they can be informed of the results of the test via E-mail or mail. Following the study, they had this chance to continue the therapy sessions alone. After the end of the therapy sessions of the two experimental groups, control group that was waiting for therapy received family therapy sessions.

***Measurement Instrument***

*Differentiation of Self Iventory-2 (DSI-2): *The DSI (13) is a 43-item questionnaire ranging from 1 (not all true for me) to 6 (very true of me) using 6-point scales. The DSI contains four subscales: emotional reactivity (ER = 11 items), "I" position (ID = 11 items), emotional cut-off (EC = 13 items), and fusion with others (FO = 8 items). Confirmatory Factor Analysis (CAF), by Skowron (13) has confirmed the mentioned subscales. The reliability of the questionnaire was calculated by internal consistency using Cronbach’s alpha. For ER, ID, EC and FO, the calculated values were, respectively 0.89, 0.81, 0.84 and 0.86 (35). In the present study, this questionnaire was translated into Persian and submitted to the instructors of the Counseling Department of Isfahan University, Iran in order to examine its content validity. Then, the questionnaire was tested on 40 clients (17 men and 23 women). These clients were chosen randomly from those who were referred to the counseling centers throughout Isfahan. The subjects were asked to note down whatever ambiguity or question they had about the items. Results revealed that there is no need to correct the items. At the end, the Cronbach’s alpha figures for the mentioned scales were respectively 0.89, 0.91, 0.81 and 0.86 which seems adequate for research goals.

*Couples intimacy inventory:* This questionnaire has been introduced by Olya et al. (51). It includes 85 questions in the from of Likert spectrum. It covers nine dimensions of marital intimacy: emotional intimacy (11 predicates), intellectual intimacy (8 predicates), physical intimacy (6 predicates), social-recreational intimacy (8 predicates), communication intimacy (11 predicates), spiritual intimacy (9 predicates), psycological intimacy (9 predicates), sexual intimcacy (8 predicates), and general intimacy (15 predicates). In this research, general intimacy was used. To determine content validity, 5 family counseling experts were used to give their comments (52). To determine its reliability, Cronbach’s alpha was used and total Cronbach’s alpha was calculated as 98.58. In this research, in initial study, reliability of the inventory by internal consistency method (Cronbach’s alpha) was 0.86.

To analysis the data, descriptive statistics methods (means and standard deviations (SD) and inferential statistics methods including multivariate analysis of covariance (MANCOVA), analysis of variance (ANOVA), and Bonferroni test were used. 

## Results

Table 1 shows summary of the subjects' demographic variables.

Table 2 shows that means (SD) of self-differentiation and general intimacy were different in three phases of the study (pre-test, post-test and follow-up).

The results presented in table 3 indicate that according to the post-test outcomes, there was a significant difference between the groups in terms of means of self-differentiation and its subscales (emotional reactivity, “I” position, emotional cut-off and fusion with others) and general intimacy (p < 0.0001, F= 46.09). However, The results showed that there was also a significant difference between PCT and REBT on post-test regarding means of the subscales (emotional reactivity, “I” position, emotional cut off and fusion with other) and general intimacy for group.

**Table 1:** Summary of the demographic variables of the studied sample

**Variables** **Index**		**Males (N = 11)**	**Females (N = 31)**
**Age**	Mean	35.2	33.1
**Education**	Below Junior high	41%	51%
	High school	33%	44%
	Bachelor	22%	03%
	Master or Doctor	00.4%	02%
**Employed**	Yes	65%	09%
	No	35%	91%
**Length of marriage (year)**	Mean	8	9
	Range	1-15	2-17
**Income (US$/month)**	Mean	301.34	54.23
**Current living situation**	Live with spouse	21%	14%
	Live with parents	06%	84%
Live alone	73%	02%

**Table 2:** Means and standard deviations of self-differentiation and its subscales and general intimacy

**Statistics**		**Means ± SD**		
**Groups**		**Pre-test**	**Post-test**	**Follow-up**
**Person-Centered Therapy**	Self- Differentiation	214.34 ± 13.83	87.45 ± 13.61	84.67 ± 14.28
	Emotional reactivity	52.23 ± 9.28	18.78 ± 8.48	21.57 ± 7.35
	“I” position	49.33 ± 10.29	17.56 ± 8.65	19.34 ± 8.57
	Emotional cut off	57.12 ± 8.18	23.45 ± 6.98	21.67 ± 8.89
	Fusion with other	39.34 ± 12.32	15.12 ± 11.76	13.35 ± 9.09
	General intimacy	75.12 ± 14.54	36.19 ± 13.43	33.87 ± 15.15
**Rational-Emotive-Behavior Therapy**	Self- Differentiation	201.45 ± 11.61	81.54 ± 14.23	79.32 ± 12.49
	Emotional reactivity	57.34 ± 8.17	21.43 ± 9.59	18.89 ± 9.09
	“I” position	47.21 ± 11.31	16.98 ± 7.35	20.01 ± 14.17
	Emotional cut off	55.35 ± 9.56	26.06 ± 9.11	24.61 ± 11.35
	Fusion with other	37.25 ± 17.43	14.42 ± 10.47	16.11 ± 12.11
	general intimacy	72.67 ± 15.46	34.18 ± 12.68	37.54 ± 10.38
**Control**	Self- Differentiation	201.45 ± 14.67	197.02 ± 16.35	213.03 ± 17.06
	Emotional reactivity	57.34 ± 11.28	54.11 ± 12.78	59.43 ± 14.12
	“I” position	47.21 ± 15.54	52.45 ± 13.67	56.34 ± 17.14
	Emotional cutoff	55.35 ± 12.43	52.06 ± 14.34	53.32 ± 13.45
	Fusion with other	37.25 ± 16.49	32.89 ± 17.45	39.08 ± 16.66
	general intimacy	72.87 ± 13.21	35.85 ± 11.21	32.05 ± 15.87

**Table 3:** Multi-analysis of covariance (MANCOVA) on post-test means of self- differentiation and its subscales (emotional reactivity, “I” position, emotional cut-off and fusion with others)

**Variables**	**Name of test**	**Values**	**df error**	**df Hypoth**	**F**	**p**	**Eta Squared**	**Observation Power**
**Self- Differentiation**	Wilks' Lambda	0.783	047	6	0.214	0.8190	0.040	0.12
**pre** **-** **test, Emotional reactivity**	Wilks' Lambda	0.872	047	6	0.412	0.6430	0.030	0.09
**pre** **-** **test, “I” position**	Wilks' Lambda	0.432	047	6	1.030	0.1780	0.091	0.23
**pre** **-** **test, Emotional cut off**	Wilks' Lambda	0.213	047	6	1.880	0.1280	0.030	0.56
**pre** **-** **test, Fusion with other**	Wilks' Lambda	0.902	047	6	1.320	0.3410	0.130	0.34
**pre** **-** **test, ** **general intimacy**	Wilks' Lambda	0.734	047	6	0.745	0.5630	0.210	0.52
**Groups**	Pillai's Trace	0.704	107	6	8.450	0.0001	0.460	1.00
	Wilks' Lambda	0.074	103	6	46.09	0.0001	0.810	1.00
	Hotelling's Trace	9.230	08	6	36.12	0.0001	0.790	1.00
	Roy's Largest Root	9.450	047	3	125.1	0.0001	0.890	1.00

**Table 4:** Multi-analysis of covariance (MANCOVA) on post-test means of self- differentiation and its subscales (emotional reactivity, “I” position, emotional cut-off and fusion with others) and general intimacy

**Variables**	**Source variable**	**Sum** **Squared**	**df**	**Means** **Squared**	**F**	**P**	**Eta Squared**	**Observation Power**
**Self- Differentiation**	pre-test	009.34	01	0008.53	000.436	0.3240	67	0.01
	Group	6432.23	02	3613.17	167.340	0.0001	1.00	0.92
	Error	0743.64	47	0013.35	0000.0001			
**Emotional reactivity**	pre-test	0007.12	01	0006.31	000.315	0.2390	89	0.01
	Group	4211.12	02	1502.28	143.450	0.0001	1.00	0.86
	Error	0531.23	47	0011.02	0000.0001			
**“I” position**	pre-test	0005.25	01	0007.73	000.421	0.3480	74	0.01
	Group	5326.46	02	2419.06	174.160	0.0001	1.00	0.87
	Error	0653.34	47	0012.13	0000.0001			
**Emotional cut off**	pre-test	0004.73	01	0005.94	000.741	0.5040	91	0.01
	Group	6149.67	02	2132.43	214.160	0.0001	1.00	0.93
	Error	0543.68	47	0009.67	0000.0001			
**Fusion with others**	pre-test	0004.98	01	0006.31	000.523	0.4150	86	0.01
	Group	5948.21	02	2311.04	232.360	0.0001	1.00	0.91
	Error	0439.08	47	0016.21	0000.0001			
**General intimacy**	pre-test	0007.65	01	0006.74	000.547	0.3480	64	0.01
	Group	6856.42	02	5347.34	136.420	0.0001	1.00	0.94
	Error	0615.25	47	0031.26	0000.0001			

The results presented in table 4 indicate that on post-test analyses, means of self- differentiation and its subscales and general intimacy for groups, a significant difference was found (p < 0.001).

The results presented in table 5 indicate that Bonferroni post-hoc test was employed to compare the means of self-differentiation and its subscales and general intimacy in various pairs of groups. There was a significant difference between PCT and REBT with control group.

**Table 5:** The results of Bonferroni post-Hoc test to compare means of self- differentiation and its subscales(emotional reactivity, “I” position, emotional cut-off, and fusion with others) and general intimacy among the three studied groups

**Variables**	**Groups**	**Means**	**PCT** †	**REBT** ‡	**Control**
**Self- differentiation**	PCT†	21.34	ــــ	(p < 0.005)	(p < 0.003)
	REBT‡	17.45	(p < 0.004)	ــــ	(p < 0.003)
	Control	43.21	(p < 0.001)	(p < 0.001)	ــــ
**Emotional reactivity**	PCT †	17.06	ــــ	(p < 0.005)	(p < 0.005)
	REBT ‡	13.56	(p < 0.003)	ــــ	(p < 0.001)
	Control	42.56	(p < 0.001)	(p < 0.001)	ــــ
**“I” position**	PCT †	19.79	ــــ	(p < 0.001)	(p < 0.001)
	REBT ‡	16.49	(p < 0.005)	ــــ	(p < 0.001)
	Control	41.61	(p < 0.001)	(p < 0.005)	ــــ
**Emotional cut off**	PCT †	16.83	ــــ	(p < 0.001)	(p < 0.004)
	REBT ‡	15.06	(p < 0.005)	ــــ	(p < 0.001)
	Control	39.32	(p < 0.001)	(p < 0.001)	ــــ
**Fusion with others**	PCT †	18.36	ــــ	(p < 0.005)	(p < 0.005)
	REBT ‡	17.84	(p < 0.001)	ــــ	(p < 0.001)
	Control	37.73	(p < 0.005)	(p < 0.001)	ــــ
**General intimacy**	PCT †	21.34	ــــ	(p < 0.005)	(p < 0.005)
	REBT ‡	19.49	(p < 0.001)	ــــ	(p < 0.001)
	Control	42.57	(p < 0.005)	(p < 0.001)	ــــ

† Person-Centered Therapy;

‡ Rational, Behavioral and Emotive Therapy

**Table 6:** Comparing mean differences of post-test and follow-up scores on dependant variables of self-differentiation and its subscales (emotional reactivity, “I” position, emotional cut-off and fusion with others) and general intimacy through the t test

**Dependent Variable**	**Groups**	**DF**	**Post-test**	**Follow-up**	**T- test** **(Post- Follow test)**	**p**
**Self- Differentiation**	PCT†	1	087.45	084.67	02.78	00.3400
	REBT‡	1	018.78	021.57	02.79	0.317
	Control	1	017.56	019.34	01.78	0.632
**Emotional reactivity**	PCT †	1	023.45	021.67	01.78	0.701
	REBT‡	1	015.12	013.35	01.77	0.462
	Control	1	036.19	033.87	02.32	0.138
**“I” position**	PCT †	1	081.54	079.32	02.22	0.627
	REBT‡	1	021.43	018.89	02.54	0.784
	Control	1	016.98	020.01	03.03	0.432
**Emotional cut off**	PCT †	1	026.06	024.61	01.45	0.226
	REBT‡	1	014.42	016.11	01.69	0.413
	Control	1	034.18	037.54	03.36	0.514
**Fusion with other**	PCT †	1	197.02	213.03	16.01	0.852
	REBT‡	1	054.11	059.43	05.32	0.321
	Control	1	052.45	056.34	03.36	0.902
**General intimacy**	PCT †	1	052.06	053.32	01.26	0.784
	REBT‡	1	032.89	039.08	06.19	0.534
	Control	1	035.85	032.05	03.08	0.854

† Person-Centered Therapy;

‡ Rational, Behavioral and Emotive Therapy

The results presented in table 6 indicate that there was not a significant difference inpost-test and follow-up stages of the study between PCT and REBT. However, there was not any significant difference in the other two phases of the study (post-test and follow-up) between PCT and REBT. 

## Discussion

The results obtained here are in agreement with previous reports about the effectiveness of the cognitive therapy method on improving the implicated variables (32-35). The current study is in conformity with other studies such as Timm (36), Skowron (31), Hobby (37), Baum and Shnit (38), Teasing (21), and Hollander (39). The results of this research are in accordance with the theoretical findings and concepts of this approach, since Ellis approach is, in fact, a direct method and sometimes a therapist moves ahead of the client and uses verbal shock. Based on clinical experiences, it seems 

that especially in the culture of Kurdistan province, Iran this method is more effective on self-differentiation and its subscales (41). Studies by Elieson (33) are cases in point. Persuading the clients to be active is more important than that we can stimulate the clients. The current study is in conformity with other studies such as Timm (36), Skowron (31), Hobby (37), Baum and Shnit (38), Teasing (21), and Hollander (39). 

Although the results show that both Ellis’s cognitive therapy method and client-centered therapy method have been effective on self-differentiation and its subscales, it can be assumed that self-differentiation individuals suffer from cognitive problems such as irrational thinking or that they have a biological tendency of self-destruction. According to the present study Ellis’s cognitive approach sought to change the individuals’ irrational attitude about the outside event to rational attitude so that its behavioral consequences could be changed. Since according to Roger and Ellis’s approach both anxiety therapies have been successful, the results of the curent study is in conformity with studies conducted by Bartle-Haring and Gregory (25) Killen and Wainryb (24), and Peleg-Popko (26). 

The results of the study point to several issues related to prevention and intervention of psychological distress for Iranian clients and couples. There was valid evidence that adjustment difficulties or marital conflict may be a sign of underlying differentiation in couples. Rather than simply treating adjustment problems, therefore, therapists may need to focus on how poor health status can influence psychological conflicts. Although Iranian individuals are considered collectivistic and thus it is necessarily encourage individuals to achieve self-differentiation, and psychological rational and empathy with another, we believe that there is a valid need for Iranian individuals to strive for empathy and self-differentiation. As noted by Rogers, in a collectivist culture, respect for the process of actulity a self means working with, not against, the individuals's values and norms (3). Indeed, in such a situation, clinicians need to be very attentive to the fact that the martial conflict process in the Iranian divorce clients is very different from other cultures. In order to increases therapeutic effects clients and couples who experience marital conflict, counselors and psychotherapists need to make effort to increase process of rationnal and understand of empathy which is closely related to well-individual functioning. In collectivistic and hierarchical Iranian culture, individuals functioning level can be improved when therapists who use the Ellis and Rogers approaches focus on rationnal processes and understand empathy and thus protect the dignity of the individual and honors the good name of the divorce clients. We expect that the results of the present study would have meaningful implications for the prevention and treatment of individuals and families. Future research on this issue should include several type participants and include individual measures to discern whether the similarities and differences found in the present study result from individual level of value orientation or from belonging to specific belief. It will be also valuable to examine the problem belief in the relationship of individual functioning with self and another, since high process of rationnal and understand of self-differentiation are assumed to be predicated on family functioning and the definition of marital conflict functioning may be different across different cultures.

In sum, it can be stated that the present study’s hypotheses were confirmed. The current study was a valuable study of its kind in the examination of the effects of counseling therapy, especially person-centered methods and rational-emotive-behavioral approach on self-differentiation. It is hoped that the findings of the current study be noted in Iran and other parts of the world so that counselors and therapists would be able to choose appropriate approaches for family behavioral problems in any fields and achieve therapy aims. Thus, using this approach to modify processing of excitements of couples and improving their emotional skills, intimacy and communication can be effective. 
